# Human interleukin-12α and EBI3 are cytokines with anti-inflammatory functions

**DOI:** 10.1126/sciadv.adg6874

**Published:** 2023-10-25

**Authors:** Karen Hildenbrand, Sina Bohnacker, Priyanka Rajeev Menon, Anna Kerle, Ulrich F. Prodjinotho, Franziska Hartung, Patrick C. Strasser, Dragana A. M. Catici, Florian Rührnößl, Martin Haslbeck, Kathrin Schumann, Stephanie I. Müller, Clarissa Prazeres da Costa, Julia Esser-von Bieren, Matthias J. Feige

**Affiliations:** ^1^ Center for Functional Protein Assemblies (CPA), Department of Bioscience, TUM School of Natural Sciences, Technical University of Munich, 85748 Garching, Germany.; ^2^ Center of Allergy and Environment (ZAUM), Technical University of Munich and Helmholtz Zentrum München, 80802 Munich, Germany.; ^3^ Institute for Microbiology, Immunology and Hygiene, Technical University of Munich, 81675 Munich, Germany.; ^4^ Center for Global Health, Technical University of Munich, 81675 Munich, Germany.; ^5^ German Center for Infection and Research (DZIF), partner site Munich, Germany.; ^6^ Department of Immunobiology, Université de Lausanne, 1066 Epalinges, Switzerland.

## Abstract

Interleukins are secreted proteins that regulate immune responses. Among these, the interleukin 12 (IL-12) family holds a central position in inflammatory and infectious diseases. Each family member consists of an α and a β subunit that together form a composite cytokine. Within the IL-12 family, IL-35 remains particularly ill-characterized on a molecular level despite its key role in autoimmune diseases and cancer. Here we show that both IL-35 subunits, IL-12α and EBI3, mutually promote their secretion from cells but are not necessarily secreted as a heterodimer. Our data demonstrate that IL-12α and EBI3 are stable proteins in isolation that act as anti-inflammatory molecules. Both reduce secretion of proinflammatory cytokines and induce the development of regulatory T cells. Together, our study reveals IL-12α and EBI3, the subunits of IL-35, to be functionally active anti-inflammatory immune molecules on their own. This extends our understanding of the human cytokine repertoire as a basis for immunotherapeutic approaches.

## Teaser

The human cytokine repertoire has more members than previously thought which limit inflammatory responses.

## INTRODUCTION

Interleukins (ILs) are key signaling molecules of the immune system that are classified into families on the basis of structural similarities (*1*). The IL-12 family consists of at least four members—IL-12, IL-23, IL-27, and IL-35—and is assigned to one family because of its unique heterodimeric character that separates it from other ILs (*2*, *3*). Each member is composed of an α subunit that shows a cytokine-characteristic four-helix bundle fold (IL-12α/p35, IL-23α/p19, and IL-27α/p28) and a β subunit composed of two fibronectin (Fn) III domains [Epstein-Barr virus–induced gene 3 (EBI3)] with an optional immunoglobulin (Ig) domain (IL-12β/p40) (*2*, *3*). A remarkable feature of the IL-12 family is that nature uses extensive sharing of only five subunits to build the four heterodimers (IL-12, IL-12α/IL-12β; IL-23, IL-23α/IL-12β; IL-27, IL-27α/EBI3; and IL-35, IL-12α/EBI3). A similar combinatorial complexity applies to the IL-12 family receptors, which are also heterodimers formed by five different chains (IL-12Rβ1, IL-12Rβ2, IL-23R, IL-27Rα, and gp130). Binding of the suitable IL induces receptor chain dimerization, thereby activating Janus kinase–signal transducer and activator of transcription (JAK-STAT) signaling pathways (*2*, *4*).

Subunit sharing on the level of cytokines and receptors may suggest closely related functions of the IL-12 family members, but the opposite holds true. The effects of the four family members are unexpectedly diverse and even opposing. The mostly proinflammatory IL-12 and IL-23 are drivers of inflammation via T helper 1 (T_H_1) differentiation and T_H_17 development, respectively (*4*). IL-27 is an immunomodulatory cytokine that on the one hand is able to promote T_H_1 differentiation and on the other hand suppresses proinflammatory T_H_17 cells and induces anti-inflammatory IL-10–producing T regulatory (T_reg_) 1 cells (*5*). In contrast, IL-35 is the only strictly inhibitory family member and acts by suppression of conventional T cells and their conversion into induced suppressive iTr35 cells (induced T regulatory population making IL-35) (*6*).

Among the IL-12 family cytokines, IL-35 remains particularly ill-understood on a molecular level and shows unexpectedly distinct features. In contrast to the other family members, IL-35 is predominantly produced not only by subsets of T_reg_ and B cells, to a lesser extent by dendritic cells, but also by placental trophoblasts and tumor cells and is critically involved in the immunosuppressive capabilities of these different cell types (*6*–*8*). Because of its inhibitory character, IL-35 plays an important role in keeping immune reactions in check but is also associated with a broad range of different immune-related diseases. On the one hand, a variety of autoimmune, inflammatory, and allergic diseases are linked to reduced circulating IL-35 levels, underlining its importance in maintaining self-tolerance (*8*–*11*). On the other hand, IL-35 is reported to act as a driver in various cancers (*12*–*18*). In strong contrast to the detailed insights into its many biological functions stands our limited understanding of the structure and receptor repertoire of IL-35. For IL-12, IL-23, and IL-27, experimental structures and experimentally validated models of the isolated cytokines (*19*–*22*) and their receptor binding are available (*23*–*28*). In case of IL-35, however, even comprehensive studies have failed to identify its heterodimerization interface (*29*), which thus appears to be distinct from the other family members. Furthermore, several studies point toward independent functions of IL-35 subunits, but insights into their biological relevance, molecular mechanisms of secretion, and function remain very limited (*30*–*35*). Last, IL-35 signaling does not occur via a single heterodimeric receptor, as it is the case for the other IL-12 family members. Four possible receptor chain combinations (IL-12Rβ2:gp130, IL-12Rβ2:IL-12Rβ2, gp130:gp130, and IL-12Rβ2:IL-27Rα) are so far reported as IL-35 receptors (*33*, *36*). How one molecule can engage this diverse receptor repertoire remains unexplained. This pronounced discrepancy between the biological importance and our molecular understanding of IL-35 demands for further studies.

In this work, we show that IL-12α and EBI3, the subunits of IL-35, mutually promote their secretion from cells not only as a heterodimer but also as nonheterodimer. Building on this, we perform a detailed biochemical and functional analysis of human IL-12α and EBI3, which we show to be stable proteins with anti-inflammatory properties. Our findings provide important steps forward in our understanding of key human immune signaling molecules and will facilitate future clinical IL-12 family–centered approaches.

## RESULTS

### IL-12α and EBI3 mutually promote their secretion in different assembly states

**Fig. 1.**
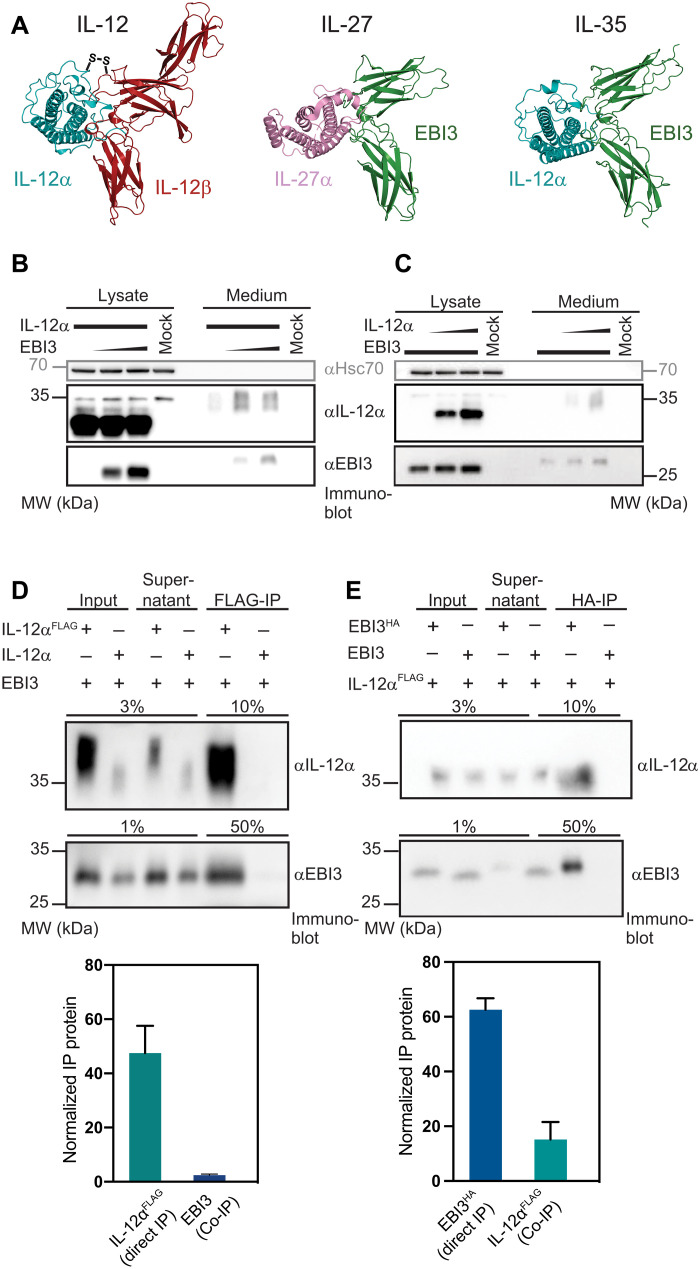
IL-12α and EBI3 mutually promote their secretion and form IL-35. (**A**) IL-35 (alphaFold2 docked model) shares its α subunit IL-12α with IL-12 [Protein Data Bank (PDB): 3HMX], and its β subunit EBI3 with IL-27 (PDB: 7u7n). To assess mutually induced secretion of IL-12α and EBI3, constant DNA amounts of IL-12α were cotransfected with increasing DNA amounts of EBI3 (**B**) or vice versa (**C**). Both IL-12α and EBI3 show a reduced mobility in the medium, indicating that both subunits are retained in the endoplasmic reticulum in isolation and traverse the Golgi during secretion, as indicated by modification of their glycans. (**D**) Co-IP of FLAG-tagged IL-12α coexpressed with EBI3 in the cell medium verifies assembly for these two proteins. Quantification of the IL-12α IP efficiency and the fraction of EBI3 that is found in complex with IL-12α is shown (*n* = 3 ± SD). (**E**) Co-IP of secreted HA-tagged EBI3 coexpressed with IL-12α^FLAG^ verifies assembly for these two proteins in the medium. Quantification of the EBI3^HA^ IP efficiency and the fraction of IL-12α^FLAG^ that is found in complex with EBI3^HA^ is shown (*n* = 5 ± SD). Constructs were expressed in human embryonic kidney (HEK) 293T cells. One representative immunoblot is shown in each case.

Since its first description (*37*) and functional characterization (*8*), IL-35 has remained a structurally ill-defined cytokine. This has hampered progress in understanding this potent immunosuppressive molecule and in potentially using or targeting it in the clinics. IL-35 is composed of IL-12α and EBI3, subunits that are shared with IL-12 or IL-27, respectively (Fig. 1A). In agreement with a productive interaction of IL-12α and EBI3, IL-12α was hardly secreted in isolation but required the presence of EBI3 to induce its secretion (Fig. 1B). Vice versa, IL-12α slightly increased EBI3 secretion (Fig. 1C). This dependency hints toward formation of a complex between both subunits. In accordance with the first report on IL-35 (*37*), we were able to detect interaction of both subunits in coimmunoprecipitation (co-IP) experiments in cell lysates (fig. S1) and when secreted into the cell medium (Fig. 1, D and E). However, our data revealed that only 2% of the overall secreted EBI3 pool coimmunoprecipitated with IL-12α-FLAG (for which the IP efficiency was 48%; Fig. 1D). Vice versa, only 15% of the secreted IL-12α pool coimmunoprecipitated with secreted EBI3-HA (for which the IP efficiency was 63%; Fig. 1E). Assuming an IP efficiency of 100%, our data thus show that when coexpressed, only approximately 4% of secreted EBI3 were bound to IL-12α, and only approximately 24% of secreted IL-12α were complexed with EBI3. Thus, at first glance, IL-35 shares key features of other IL-12 family members: assembly-induced secretion of the subunits and their interaction, albeit weak (*3*, *37*–*40*). In contrast to other IL-12 family members, however, most of the secreted subunits do not seem to be part of heterodimeric IL-35.

**Fig. 2.**
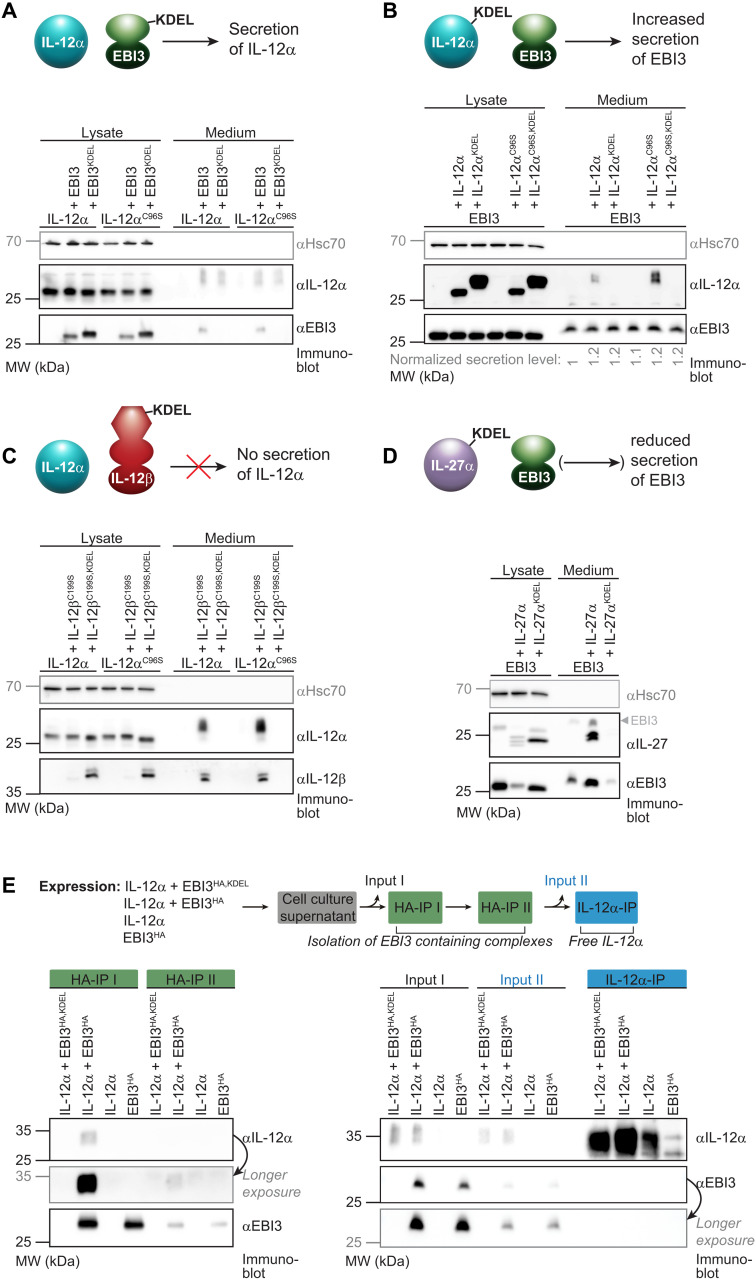
IL-35 subunits can be secreted as nonheterodimers in contrast to IL-12 and IL-27. (**A**) EBI3 induces the secretion of IL-12α even when it is retained in the ER (via a C-terminal KDEL sequence). EBI3-induced secretion is observed for wild-type IL-12α and a variant lacking the cysteine that forms an interchain disulfide bond in IL-12 (C96S). (**B**) The same as in (A), only that IL-12α was furnished with a KDEL ER-retention sequence and secretion of EBI3 was monitored. EBI3 secretion was slightly increased by coexpression with both IL-12α and the C96S variant. Quantifications of EBI3 secretion are shown below the blot. (**C**) A similar analysis for IL-12 reveals that IL-12β^C199S^, with an ER retention sequence, does not induce secretion of free IL-12α but instead co-retains it in the cell. The same is observed for the combination of IL-12α^C96S^ with IL-12β^C199S^, with both proteins lacking the cysteines that form the interchain disulfide bond in IL-12. (**D**) Coexpression of wild-type EBI3 and IL-27α leads to the secretion of IL-27. When IL-27α was ER-retained (IL-27α^KDEL^), EBI3 secretion was reduced, in contrast to (B). (**E**) Free IL-12α can be detected in medium samples after pulldown of EBI3. IL-12α and EBI3 are cotransfected, and cell supernatants underwent two consecutive HA-IPs to isolate EBI3^HA^-containing complexes. IL-12α is also coimmunoprecipitated as can be seen in the IL-12α blot after HA-IP I and II, indicating pulldown of IL-35. The final IL-12α–IP reveals remaining IL-12α in the medium that is not interacting with EBI3. Band intensities show a significantly higher amount of free IL-12α when coexpressed with EBI3 (or EBI3^KDEL^) compared to transfection in isolation without EBI3.

This prompted us to more thoroughly investigate IL-12α and EBI3 secretion upon coexpression. These analyses revealed a fundamentally different behavior of the IL-12α:EBI3 pairing in comparison to other IL-12 family members. When either IL-12α or EBI3 were furnished with a C-terminal KDEL sequence (IL-12α^KDEL^ or EBI3^KDEL^, respectively), which leads to endoplasmic reticulum (ER) retention of the respective protein (*41*), these subunits were retained in cells (Fig. 2, A and B), as expected. Quite unexpectedly, though, EBI3^KDEL^ could still induce secretion of IL-12α (Fig. 2A). Furthermore, levels of secreted IL-12α appeared relatively independent of the cosecretion of EBI3 (Fig. 2A). Likewise, IL-12α^KDEL^ slightly increased secretion of EBI3 over its basal levels (Fig. 2B). These findings show that IL-12α and EBI3 mutually promote their secretion without necessarily being secreted as a heterodimer. This behavior is in contrast to other known pairings within the IL-12 family that share subunits with IL-35. For IL-12, where IL-12α pairs with IL-12β, IL-12β^C199S,KDEL^ did not induce the secretion of IL-12α, even when the interchain-disulfide bond that is dispensable for IL-12 secretion (*42*) was deleted (C96S in IL-12α and C199S in IL-12β; Fig. 2C). Instead, IL-12β^C199S,KDEL^ co-retained IL-12α in cells (Fig. 2C). Analogously, for IL-27, where EBI3 pairs with IL-27α, IL-27α^KDEL^ did not increase the secretion of EBI3 but instead reduced its secretion and thus also co-retained it (Fig. 2D). Furthermore, EBI3^KDEL^ has recently been shown to co-retain IL-27α in cells (*43*). Together, these data reveal a different behavior for the IL-12α:EBI3 pairing than for other subunit combinations within the IL-12 family. IL-12α and EBI3 may thus not only be secreted in an assembled state as IL-35, but also as free IL-12α and EBI3, which would also explain the low amount of complex formation observed in cell media (Fig. 1, D and E). To further test our hypothesis of a separate IL-12α and EBI3 secretion in cells expressing both subunits, we next examined whether free IL-12α and EBI3 were also detected in the medium of coexpressing cells when none of the subunits contained an ER retention sequence. In addition to assembled IL-35, our experiments confirmed the presence of secreted, unassembled EBI3 (Fig. 2E). Furthermore, we could detect a pool of free IL-12α secreted from IL-12α and EBI3 coexpressing cells (Fig. 2E). Thus, together, our data show that cells expressing IL-12α and EBI3 secrete not only assembled IL-35 but also an excess of unassembled IL-12α and EBI3.

### IL-12α and EBI3 are stable proteins in isolation

Our findings suggest that IL-12α and EBI3 may act as independent immune signaling molecules secreted from cells that produce both subunits. This notion would have a profound impact on our understanding of the setup of the human IL-12 family and thus requests further analyses on the structure and function of isolated IL-12α and EBI3.

**Fig. 3.**
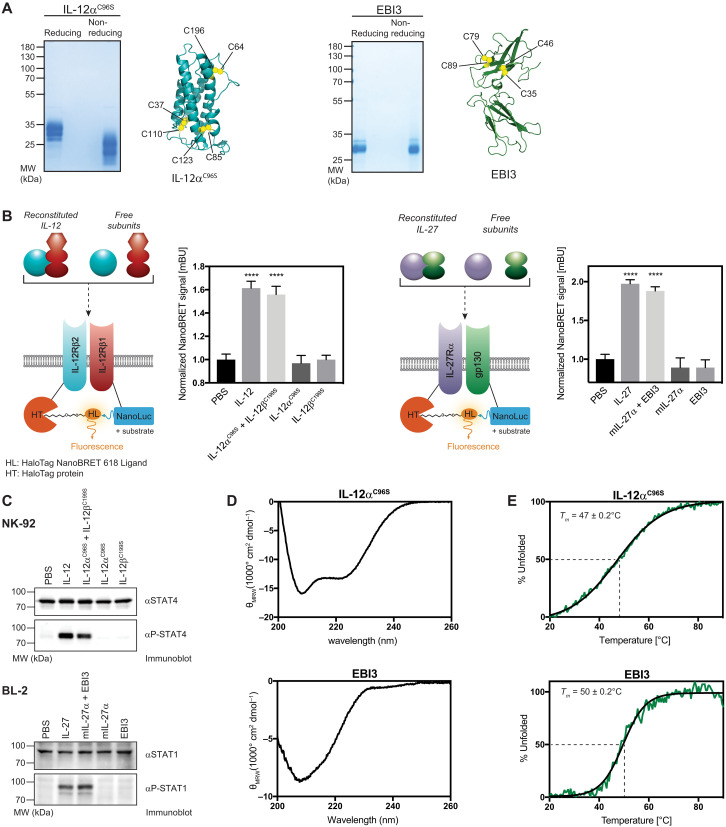
Recombinant human IL-12α^C96S^ and EBI3 are stable and well-structured proteins. (**A**) Analysis of purified IL-12α^C96S^ and EBI3 by reducing and nonreducing SDS–polyacrylamide gel electrophoresis (SDS-PAGE). Faster migration on nonreducing SDS-PAGE indicates the presence of disulfide bonds. Positions of intramolecular disulfide bonds are indicated in each subunit structure. (**B**) Reconstituted IL-12 and IL-27 are able to induce receptor heterodimerization. COS-7 cells were cotransfected with the indicated receptor chains equipped with the NanoBRET reporter system. Cells were stimulated with purified IL-12α^C96S^, which was previously incubated with recombinant human IL-12β^C199S^, or EBI3, previously incubated with murine IL-27α or the isolated subunits as indicated (10 nM final concentrations). Graphs represent the normalized NanoBRET signal (*n* = 3 ± SD). Statistical significance was calculated by one way analysis of variance (ANOVA) followed by Dunnett’s multiple comparisons test; ****P < 0.0001 compared with the corresponding phosphate-buffered saline (PBS) control. (**C**) NK-92 or BL-2 cells were stimulated with preincubated IL-12α^C96S^ + IL-12β^C199S^ or EBI3 + mIL-27α or the heterodimeric cytokines (10 ng/ml final concentrations). Downstream signaling was detected by STAT-phosphorylation via immunoblot. (**D**) Far-UV CD spectra for IL-12α^C96S^ and EBI3. (**E**) IL-12α^C96S^ unfolds cooperatively with an apparent melting temperature of 47 ± 0.2°C and EBI3 with an apparent melting temperature of 50 ± 0.2°C (green line, experimental data; black line, Boltzmann sigmoidal nonlinear curve fit; transitions were not reversible).

IL-12α and EBI3 are both disulfide-containing glycoproteins (*44*), which thus require mammalian cells for protein production to obtain authentic posttranslational modifications. IL-12α, however, is mostly retained in human cells in isolation (*38*, *42*), and EBI3 is poorly secreted (*45*) (see also Fig. 1). We thus devised strategies that allowed the production of IL-12α and EBI3 in sufficient quantities from mammalian cells. As opposed to IL-35, IL-12, which also contains IL-12α (Fig. 1A), is secreted efficiently and can be purified from cell medium. We thus developed a protocol to purify IL-12 lacking the intermolecular disulfide bridge from human cells and subsequently separated IL-12α^C96S^ from IL-12β^C199S^ (fig. S2A). Notably, C96 was dispensable for EBI3-induced secretion of IL-12α (Fig. 2A). Our approach gave rise to pure and glycosylated IL-12α^C96S^ containing its intramolecular disulfide bonds (Fig. 3A). Analogously, to produce EBI3, we developed a strategy to purify IL-27 and separate EBI3 from IL-27α^L162C^, a stabilized IL-27α mutant (Fig. 3A and fig. S2B) (*43*). In both cases, our strategy allowed us to produce milligram quantities of glycosylated pure protein from mammalian cells. IL-12α^C96S^ produced in this manner remained assembly competent with IL-12β, and EBI3 remained assembly-competent with IL-27α (fig. S2C). Furthermore, reassembled IL-12 and IL-27 were biologically active and could induce receptor dimerization in a NanoBRET assay that we established for this purpose (Fig. 3B) and signaling in NK-92 or BL-2 cells, respectively (Fig. 3C). Together, this provides strong evidence that our protocol yielded properly structured proteins. Far-ultraviolet (UV) circular dichroism (CD) spectroscopy confirmed this notion and revealed an α-helical structure for IL-12α^C96S^ as expected from the crystal structure of the IL-12 molecule (*19*). EBI3 showed the far-UV CD spectroscopic signature of a β sheet protein, containing some flexible regions, in agreement with available IL-27:receptor structures (Fig. 3D) (*25*–*28*). Apparent melting temperatures were 47 ± 0.2°C (IL-12α^C96S^) and 50 ± 0.2°C (EBI3), respectively (Fig. 3E). Thus, IL-12α^C96S^ and EBI3 are both relatively stable proteins, which analytical ultracentrifugation revealed to be mostly monomeric with some self-assembly observed for EBI3 (fig. S3A). Unexpectedly, although IL-12α^C96S^ and EBI3 were correctly structured, both proteins appeared not to assemble with each other to a detectable amount in vitro as judged by analytical ultracentrifugation, co-IP, and hydrogen-deuterium exchange mass spectrometry experiments (fig. S3, A to C). Together, these detailed biophysical and functional analyses revealed that IL-12α^C96S^ and EBI3 can be purified as stable, well-folded proteins but that IL-35, in contrast to IL-12 and IL-27, cannot be readily formed from its subunits in vitro. This adds even more importance to our findings that IL-12α and EBI3 can be secreted individually and puts these as independent immune proteins on the agenda. Thus, we continued with functional studies on IL-12α and EBI3.

### IL-12α and EBI3 have anti-inflammatory properties

**Fig. 4.**
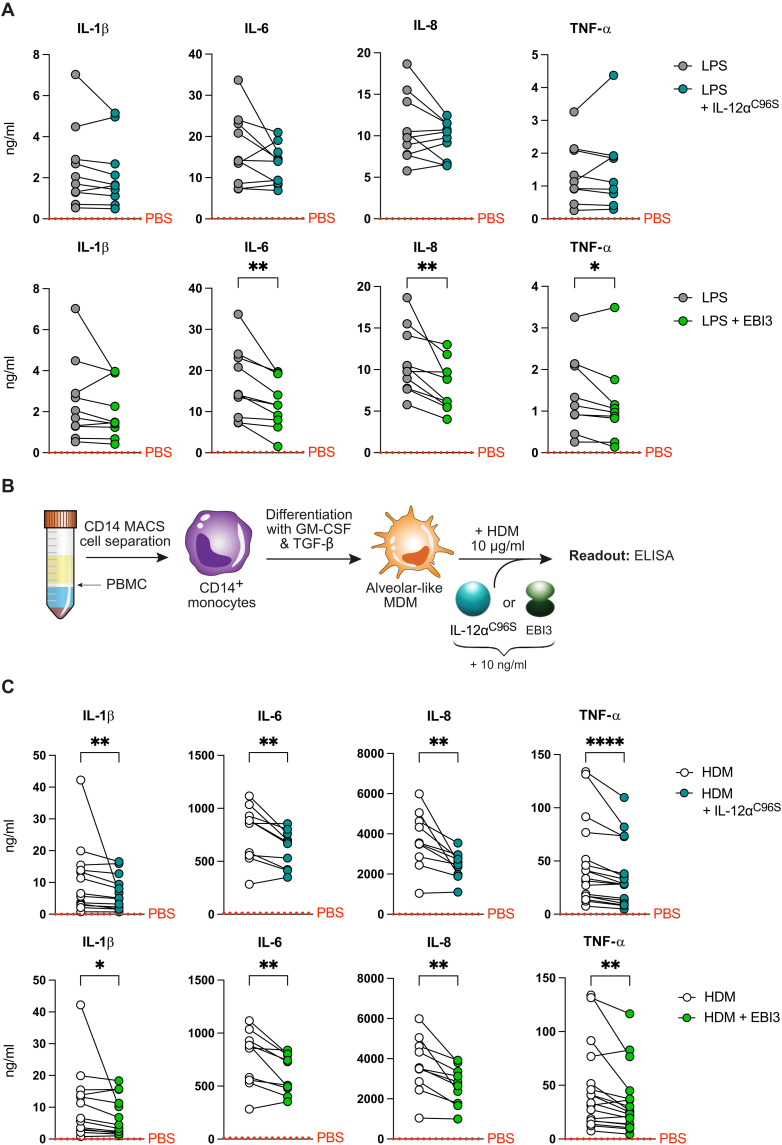
IL-12a^C96S^ and EBI3 act as immunosuppressors. (**A**) Concentrations of secreted IL-1β, IL-6, IL-8, and TNF-α [enzyme-linked immunosorbent assay (ELISA)] in supernatants from LPS stimulated human PBMCs after stimulation with IL-12a^C96S^ or EBI3 (*n* = 10 donors). (**B**) Scheme of the experimental workflow to assess IL-12a^C96S^ and EBI3 effects on alveolar like monocyte-derived macrophages (MDMs). (**C**) Amounts of IL-1β, IL-6, IL-8, and TNF-α (ELISA) produced by human MDMs (*n* = 10 to 17 donors) in supernatants after stimulation with HDM and IL-12a^C96S^ or EBI3. Dotted line indicates mean secretion of PBS-treated PBMCs or MDMs. Data are presented as individual values. Donor-dependent effect is shown by connecting line. Statistical significance was determined by Wilcoxon test. *P < 0.05, **P < 0.01, ***P < 0.001, and ****P < 0.0001.

IL-35 is described as an immunosuppressive cytokine that affects signaling in various cell types (*6*). Because our data show secretion of free IL-12α and EBI3 from cells producing both of these subunits, we wondered whether immunological effects could also be exerted by them as recent studies indicate (*30*–*33*). Pure human IL-12α and EBI3 produced in mammalian cells have not yet been investigated in this regard but now were available to us. To assess functions of IL-12α^C96S^ and EBI3, we used primary human peripheral blood mononuclear cells (PBMCs) to study effects on cells of the innate and adaptive immune system. Because IL-35 acts as an immunosuppressor, we focused on anti-inflammatory effects of its subunits IL-12α^C96S^ and EBI3. Thus, we treated PBMCs with lipopolysaccharide (LPS) to induce inflammatory responses and tested for effects of both IL-12α^C96S^ and EBI3. In agreement with a globally anti-inflammatory role, the transcription of the proinflammatory cytokines *IL1B*, *IL6*, *CXCL8*, and *TNFA* was reduced by both IL-12α^C96S^ and EBI3, whereas transcription of the immunosuppressive cytokine *TGFB* was unaffected (fig. S4A). Commercially available IL-35 showed similar effects on reducing *IL6* and *TNFA* transcription, although the effects were less pronounced than for our well-characterized IL-12α^C96S^ and EBI3 proteins (fig. S4B). When added simultaneously, IL-12α^C96S^ and EBI3 had similar effects on *IL6* and *TNFA* transcription like when added individually (fig. S4B). In overall agreement with these transcriptional effects, in addition, the secretion of proinflammatory cytokines was significantly down- regulated by EBI3, while effects of IL-12α^C96S^ were less pronounced (Fig. 4A).

As we found proinflammatory cytokines of the monocyte/macrophage compartment to be affected by IL-12α^C96S^ and EBI3, we decided to further investigate whether these cells were affected by both proteins. Lung macrophages are essential players in the development of allergic airway diseases such as asthma, where IL-35 (and, thus, possibly IL-12α and/or EBI3) play important roles (*46*). Therefore, to assess whether IL-12α^C96S^ and EBI3 can modulate proinflammatory cytokine production by myeloid cells, we isolated CD14^+^ monocytes from PBMCs, differentiated them into alveolar-like macrophages by stimulation with granulocyte-macrophage colony-stimulating factor (GM-CSF) and transforming growth factor–β (TGF-β; Fig. 4B) (*47*, *48*), and treated them with house dust mite extract (HDM), one of the most frequent triggers of allergic asthma. Treatment of the cells with either IL-12α^C96S^ or EBI3 after HDM stimulation revealed a significant suppression in secretion of the proinflammatory cytokines, IL-1β, IL-6, IL-8, and tumor necrosis factor–α (TNF-α; Fig. 4C). Again, effects of commercial IL-35 were less pronounced, and addition of both subunits simultaneously produced comparable outcomes to the separate addition of each of them. (fig. S4C). Thus, IL-12α^C96S^ and EBI3 might act as immunosuppressors in settings of, e.g., bacterial or allergenic challenge. To further test this notion, we compared the effects of IL-12α^C96S^ and EBI3 to those of IL-10, a well-characterized anti-inflammatory cytokine, and to IL-27, an immunomodulatory cytokine. IL-10 had qualitatively similar but quantitatively stronger immune-suppressive effects in comparison to IL-12α^C96S^ and EBI3. IL-10 reduced secretion of proinflammatory cytokines by approximately 40 to 90%, whereas IL-12α^C96S^ and EBI3 led to reductions by approximately 10 to 30% (fig. S4D). In contrast, IL-27 up-regulated secretion of several proinflammatory cytokines, which IL-12α^C96S^ and EBI3 down-regulated (fig. S4D). Together, these findings corroborate anti-inflammatory effects for IL-12α^C96S^ and EBI3. These are different for EBI3 than for IL-27, a cytokine where EBI3 serves as one subunit.

**Fig. 5.**
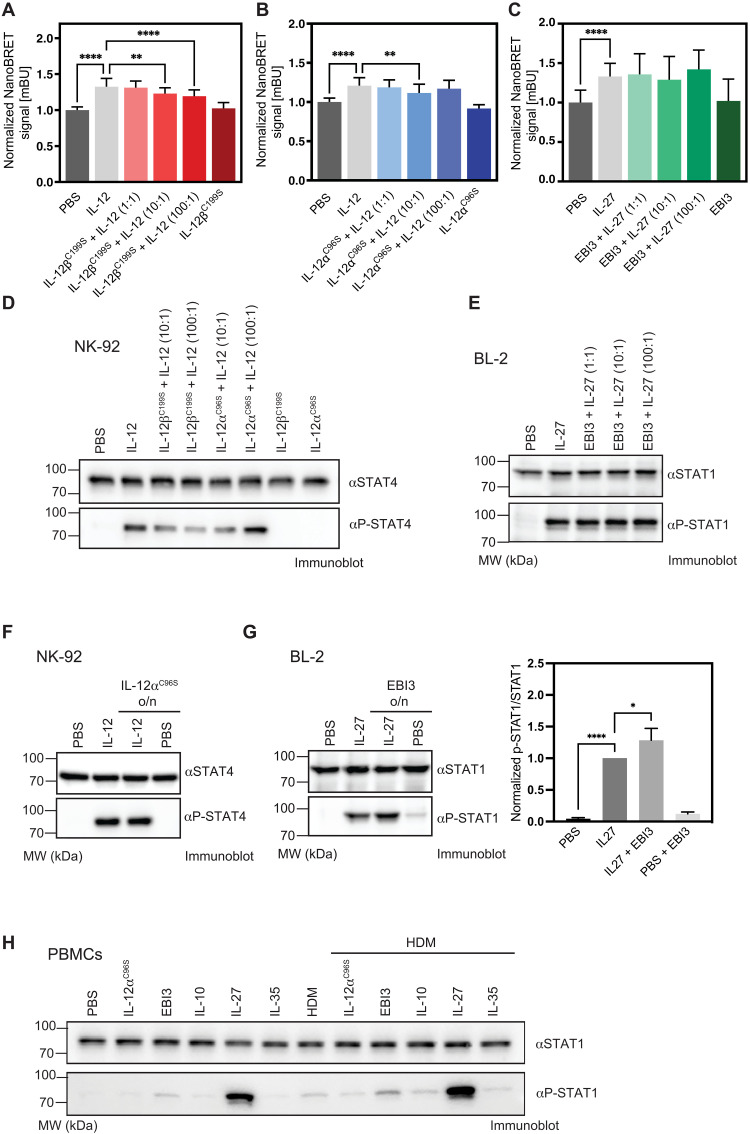
IL-12α^C96S^ and EBI3 show different signaling characteristics. (**A**) The NanoBRET reporter system indicates IL-12 receptor heterodimerization after stimulation with 1 nM IL-12, which can be blocked by a 30-min pretreatment with IL-12β^C199S^ (1, 10, or 100 nM). (**B**) In the same assay, a 30-min pretreatment with IL-12α^C96S^ (1, 10, or 100 nM) shows reduced IL-12–induced receptor heterodimerization at a 10-fold excess of IL-12α^C96S^. (**C**) A preincubation with increasing concentration of EBI3 did not have any effect on IL-27 activity. Graphs represent the normalized NanoBRET signal (*n* = 3 ± SD). (**D***)* STAT4 phosphorylation in NK-92 cells after treatment with IL-12 (1 ng/ml) is blocked by a 30-min pretreatment with IL-12β^C199S^ (10 and 100 ng/ml) or IL-12α^C96S^ (10 ng/ml). Representative immunoblots from one of three independent experiments are shown. (**E**) BL-2 cells were used to monitor IL-27 (10 ng/ml)–mediated STAT1 phosphorylation, which is not blocked by pretreatment with EBI3 (10, 100, or 1000 ng/ml) for 30 min (*n* = 3 ± SD). (**F**) NK-92 or (**G**) BL-2 cells were pretreated with IL-12α^C96S^ (10 ng/ml) or EBI3 (10 ng/ml) overnight (o/n) before addition of IL-12 or IL-27, respectively, to investigate receptor internalization. (*n* = 3 ± SD, *P < 0.05 and ****P < 0.0001) (**H**) STAT1 phosphorylation in human PBMCs treated with EBI3 and IL-12α^C96S^ compared to the cytokines IL-10, IL-27, and IL-35 and their effect after HDM stimulation. Representative immunoblots from one of three independent experiments are shown.

In the presence of proinflammatory stimuli, PBMCs and macrophages secrete IL-12 and IL-27 (*49*, *50*). Thus, it is possible that IL-12α^C96S^ inhibits proinflammatory effects mediated by IL-12 and that EBI3 does the same for IL-27 signaling. This could explain the anti-inflammatory effects that we observed for both subunits. To test for this possibility, we applied our NanoBRET reporter system. In this experiment, IL-12β, a known modulator for IL-12 signaling (*51*), inhibited IL-12–induced receptor dimerization when introduced at a 10- and 100-fold excess over IL-12 (Fig. 5A). IL-12α^C96S^ also inhibited IL-12–induced receptor dimerization at a 10-fold excess over IL-12, while at 100-fold excess, it did not (Fig. 5B). In contrast, EBI3 did not affect IL-27–induced receptor dimerization (Fig. 5C).

When we used IL-12–responsive NK-92 cells as a further test, we observed a similar reduction in IL-12–induced STAT4 phosphorylation in the presence of an excess of IL-12β^C199S^ over IL-12 (Fig. 5D). Furthermore, in line with our NanoBRET data, we observed an inhibition of STAT4 phosphorylation at a 10-fold excess of IL-12α^C96S^ over IL-12, while a 100-fold excess of IL-12α^C96S^ seemed to not affect or up-regulate phospho-STAT4 (Fig. 5D). In contrast, but similar to the NanoBRET results, a preincubation of IL-27–responsive BL-2 cells with EBI3 did not lead to reduced IL-27 signaling (Fig. 5E).

A further possibility how IL-12α^C96S^ and EBI3 could reduce signaling of the heterodimeric cytokines is by influencing receptor chain internalization, as has been described for monomeric IL-12β (*52*). To test for this possibility, we pretreated NK-92 or BL-2 cells with IL-12α^C96S^ or EBI3 overnight, respectively. After this pretreatment, the respective heterodimeric cytokines were added (IL-12 or IL-27) to NK-92 or BL-2 cells, with a further coaddition of IL-12α^C96S^ or EBI3, respectively. For IL-12α^C96S^, no effect on IL-12–induced STAT phosphorylation was observed (Fig. 5F). In contrast, overnight incubation with EBI3 itself led to a weak STAT1 phosphorylation signal in BL-2 cells that was additive with the IL-27–induced signal (Fig. 5G), but itself was not significant under the conditions tested.

On the basis of these observations, we next aimed to determine whether IL-12α^C96S^ or EBI3 can directly activate STAT signaling. In these experiments, EBI3 again induced a modest STAT1 phosphorylation, weaker than IL-27, whereas IL-12α^C96S^, IL-10, and IL-35 did not induce STAT1 phosphorylation (Fig. 5H).

Together, our data indicate that while IL-12α^C96S^ may modulate IL-12 signaling as one of its possible effects, EBI3 weakly induces tyrosine phosphorylation of STAT1. These findings led us to further study their effects on immune cell differentiation.

### IL-12α and EBI3 act as immunosuppressors by inducing regulatory T cells and reducing IL-4 production by T_H_2 cells

**Fig. 6.**
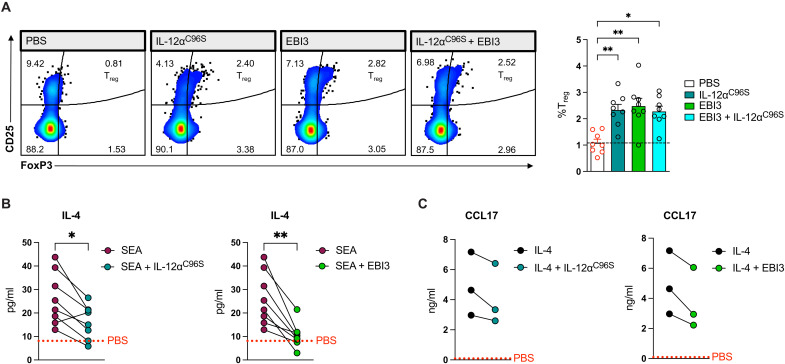
IL-12a^C96S^ and EBI3 potentiate regulatory T cell development and suppress SEA-induced IL-4 production from human PBMCs. (**A**) Percentage of CD25^hi^Foxp3^+^ T_reg_ cells [fluorescence-activated cell sorting (FACS)] in human PBMC cultures treated with IL-12a^C96S^ (20 ng/ml) and EBI3 (20 ng/ml) or the combination of both IL-12a^C96S^ and EBI3 (*n* = 8). Data are presented as means + SEM. Statistical significance was determined by Friedman test. *P < 0.05 and **P < 0.01. (**B**) Concentrations of IL-4 (ELISA) in culture supernatants from SEA-stimulated human PBMCs alone or in combination with IL-12^C96S^ or EBI3 (*n* = 8 donors). The dotted line indicates mean secretion of IL-4 from PBS-treated PBMCs. (**C**) Concentrations of CCL17 (ELISA) in culture supernatants from IL-4–stimulated human PBMCs alone or in combination with IL-12^C96S^ or EBI3 (*n* = 3 donors). (B and C) Data are presented as individual values. Donor-dependent effect is shown by the connecting line. Statistical significance was determined by Wilcoxon test. *P < 0.05 and **P < 0.01.

IL-35 was initially found to cause primary immunosuppression of effector T cell responses (*8*), and its inhibitory role is further mediated by the induction of T_reg_ cells (*53*). To investigate whether IL-12α and EBI3 can exert the same effects and thus suggest T_reg_ cells as one mediator of their suppressive properties, we assessed the potential of both subunits to potentiate these cells in human PBMC cultures. We were able to observe an induction/expansion of CD4^+^CD127^−^CD25^hi^FoxP3^+^ regulatory T cells after stimulation with both IL-12α^C96S^ and EBI3 at 20 ng/ml independent of T cell receptor stimulation or the addition of additional cytokines such as IL-2 or TGF-β (Fig. 6A and fig. S5A). This observation confirms that IL-12α and EBI3 have further immunosuppressive functions. The simultaneous addition of both subunits had comparable T_reg_-inducing effects to adding them individually (Fig. 6A). Because our data pointed toward immunological effects mediated by both IL-12α and EBI3, we aimed to further examine IL-12α and EBI3 in a disease-relevant setting using in vitro approaches. As it has been reported that IL-35 can suppress type 2 cytokine production (e.g., IL-4 and IL-5) and show a beneficial impact on allergic disease (*54*), we analyzed the capacity of its subunits to reduce type 2 cytokine production following stimulation with LPS and *Schistosoma mansoni* soluble egg antigen (SEA), a strong parasitic inflammatory trigger known to induce T_H_2 immune responses. In this setting, we observed a significant suppression of *IL-5* transcription, even at low gene expression levels, by both IL-12α^C96S^ and EBI3 in combination with LPS stimulation (fig. S5B). To further confirm this result, we incubated PBMCs with SEA and simultaneously with either IL-12α^C96S^ or EBI3. In this setting, both IL-12α^C96S^ and EBI3 reduced SEA-induced IL-4 secretion, with the effects of EBI3 being more pronounced (Fig. 6B). Moreover, both IL-12α^C96S^ and EBI3 tended to reduce the IL-4–induced secretion of CCL17, a driver of T_H_2 and T_reg_ cell recruitment (Fig. 6C) to a similar extent as IL-10, IL-27, and IL-35 (fig. S5C). These data suggest that both IL-35 subunits show clinically relevant immunosuppressive capacities in type 2 inflammatory settings.

## DISCUSSION

IL-35, which is composed of IL-12α and EBI3, is the structurally least well-defined member of the IL-12 family, and its engagement of multiple receptors has remained enigmatic. Our study now shows that cells expressing IL-12α and EBI3 do secrete not only heterodimeric IL-35 but also IL-12α and EBI3 in nonheterodimeric states, which is in contrast to other heterodimeric human IL-12 family members (*3*). In our setting, secretion of the nonheterodimeric subunits significantly exceeds secretion of IL-35, a finding that will be relevant to further test in primary immune cells. Our data revealed IL-12α and EBI3 to be stable proteins in isolation that exert anti-inflammatory effects on primary human PBMCs and macrophages under type 1 or type 2 inflammatory stimuli. HDM represents a major source of indoor allergens and is strongly associated with the development of asthma. Macrophages, as part of the innate immune system, are among the first and most abundant cells in the lung to encounter allergens and contribute to chronic airway inflammation like asthma through the production of proinflammatory cytokines (*55*, *56*). Our findings suggest that IL-12α and EBI3 may account for some of the effects in asthma that so far have been ascribed to IL-35 (*9*, *46*, *57*) and may thus be relevant to study further. One of these findings is the ability to induce regulatory T cells (*8*), which aligns well with the capacities of both EBI3 and IL-12α^C96S^ to suppress type 2 cytokines. In addition to allergens, parasitic infections trigger strong type 2 immune responses. We were able to show suppressive capacities of EBI3 and IL-12α^C96S^ on allergen-, IL-4-, and parasite-induced type 2 inflammatory responses with EBI3 exhibiting more pronounced effects. Although the effects were mild, they were even observed in a complex cell pool with only low IL-12α/EBI3 concentrations. The generally immunosuppressive functions of IL-12α and EBI3 are in agreement with previous studies (*30*, *31*, *35*, *58*). We extend these immunological data by demonstrating that these molecules are actually secreted from cells expressing both subunits and thus underline their relevance in vivo.

Our data show that at lower concentrations, IL-12α can inhibit IL-12 signaling, whereas at higher concentrations, it does not. This may be reconciled by considering the signaling capabilities of IL-12 family α subunits at higher concentrations (*43*, *59*), masking inhibitory effects. IL-12 inhibition has also recently been reported for IL-35 (*60*). Here, commercial IL-35 was used that may also contain IL-12α. In contrast, our data show that EBI3 induces weak STAT1 signaling itself but does not appear to inhibit IL-27 signaling. We thus find multiple biologically relevant signaling events to be modulated or executed by IL-12α and EBI3, which warrant further studies. These findings have several major implications for our understanding of the human IL-12 family and their role in regulating immune responses.

The independent functions of IL-12α and EBI3 suggest that some of the effects attributed to IL-35 may be exerted by its subunits, also because functional studies on IL-12 family cytokines are often performed in subunit-knockout animals so that potentially more than one factor is lost. Instability and/or low assembly propensity of IL-35 might balance effects that are either mediated by the heterodimer or the individual subunits. Recent data for IL-6 signaling consider sIL-6R and sgp130 as a systemic buffer by their ability to transiently bind IL-6, which might lead to an increased half-life and enables classical and trans-signaling (*61*, *62*). Given the structural homology between IL-12 and IL-6 family cytokines, this concept of transient interaction to regulate signaling might thus possibly need to be expanded to IL-35. Future studies will now have to show whether IL-12α and EBI3 are truly autonomous signaling molecules or can pair with other cytokines and/or shed receptor chains for signaling. Furthermore, identifying the cellular receptors will be a key next step and now will become possible using the approaches that we have developed to produce these molecules as active cytokines. Possible candidates for IL-12α signaling are IL-12Rβ2 homodimers and gp130 homodimers for EBI3 signaling, which both have been described as IL-35 receptors in the literature (*36*).

Recent studies have shown that IL-12 can be reconstituted from IL-12α and IL-12β derived from different cells (*63*), but the mechanisms of IL-12α secretion, which normally is retained in cells in isolation (*3*), had remained unclear. Our study shows how cells can secrete IL-12α and add an important further functional layer: IL-12α alone is anti-inflammatory and can potentially inhibit IL-12 signaling, yet it can pair with IL-12β to form proinflammatory IL-12, as this study and previous work have shown (*63*). The absence or presence of IL-12β–expressing cells may thus have a pronounced effect on the immune functions of adjacent cells expressing other IL-12 family subunits. In light of this finding, it is also noteworthy that IL-12–producing cells secrete an excess of free IL-12β that may inhibit IL-12 signaling, act as a signaling molecule by itself, or, as our data show, pair with IL-12α to further increase extracellular IL-12 levels. Analogously, EBI3 may have distinct effects in acting alone or pairing with other cytokines outside the cell (*35*, *63*). Thus, the combinatorial complexity of IL-12 cytokine family signaling is even larger than previously thought and may be highly context dependent.

One yet unexplained finding from our study is that cells expressing IL-12α and EBI3 secrete assembled IL-35 but that IL-12α^C96S^ and EBI3 could apparently not reconstitute IL-35 in vitro, although the subunits remained assembly competent to form other cytokines (IL-12 and IL-27) as our data show. Alternatively, complexes formed in vitro may be too unstable or of too low abundance to be detected properly. Recent work suggested CD81 to potentially assemble with IL-35 (*64*). We could not detect CD81 in complex with IL-12α and EBI3, arguing against it being an essential component of IL-35 (fig. S6). The question why IL-35 cannot be readily (or stably) formed from its subunits as our and previous work has shown (*65*) thus remains, which will be answered in future studies.

In summary, our study reveals that IL-35, in contrast to other IL-12 family members, is not secreted as a strict heterodimer, but assembly-induced folding and transient interactions enable the secretion of its subunits IL-12α and EBI3. Our discovery that IL-12α– and EBI3-producing cells thus secrete both subunits in addition to IL-35 might provide a molecular explanation for the pleiotropic signaling pathways and effects that have so far been attributed to IL-35. This is further supported by autonomous immunosuppressive properties of both subunits in primary human immune cells and their capabilities to induce regulatory T cells. Because of not only its potent immunosuppressive effects in the tumor microenvironment but also its low levels in autoimmune diseases, IL-35 is a highly attractive therapeutic target or drug candidate. While immunological effects should be reevaluated in further cell models, our data suggest that IL-12α and EBI3 should be included in this consideration, as they are secreted from cells and have anti-inflammatory effects. This may provide a basis for the development of specific antibodies against these subunits and thus opens up novel approaches for modulating immune responses in the treatment of inflammatory diseases and cancer.

### Limitations of the study

Work on IL-12α and EBI3 secretion was performed in model cell lines so that as a next step, findings need to be assessed in not only primary immune cells but also animal models.

## MATERIALS AND METHODS

### Constructs

Human IL cDNAs were obtained from OriGene (Rockville) and subsequently cloned into the pSVL vector (Amersham Biosciences). Amino acid sequences of IL-12α, IL-12β, IL-27α, and EBI3 correspond to the UniProt accession numbers P29459, P29460, Q8NEV9, and Q14213, respectively. Where indicated, constructs where C-terminally tagged with an HA or FLAG epitope-tag, separated by a (GS)_2_- or (GS)_4_-linker, or equipped with a KDEL-sequence, separated by a (GS)_3_-linker. Mutants were generated by site-directed mutagenesis. For mammalian protein purification, IL-12α^C96S^ (C-terminal TEV cleavage site followed by a GG-linker and His-tag), IL-12β^C199S^ (untagged), and IL-27α^L162C^ [C-terminal HRV-3C Protease cleavage site followed by a (GG)_2_-linker and His-tag] were cloned into the pcDNA3.4TOPO vector (Gibco) and EBI3 (untagged) into the pHEK293 ultra expression vector 1 (TAKARA). All constructs were sequenced.

### Cell culture and transient transfections

Mammalian cell experiments were performed in human embryonic kidney (HEK) 293T cells, which were cultivated in Dulbecco’s modified Eagle’s medium (DMEM) containing l-alanyl-l-glutamine (AQmedia, Sigma-Aldrich) at 37°C and 5% CO_2_. The medium was supplemented with 10% (v/v) fetal bovine serum (FBS; Gibco) and 1% (v/v) antibiotic-antimycotic solution [amphotericin B (25 μg/ml), streptomycin (10 mg/ml), and 10,000 U of penicillin; Sigma-Aldrich]. Transient transfections were performed in poly-d-lysine–coated six-well plates (Corning) using GeneCellin (BioCellChallenge) according to manufacturer’s protocol. The total transfected DNA amount was 2 μg with a DNA ratio α subunit to β subunit of 2:1 in case of IL-35 and IL-27 and 1:1 in case of IL-12. For subunit titration experiments, either 1 μg of α subunit or 1 μg of β subunit was transfected while increasing the corresponding other subunit in 0.5-μg steps. If only one subunit was transfected, then empty pSVL vector was cotransfected to maintain a total amount of 2 μg of DNA.

### Secretion experiments

Cells were transfected for 8 hours, washed twice with phosphate-buffered saline (PBS), and cultivated in 0.5 ml of complete DMEM for another 16 hours. To analyze secreted proteins, the medium was centrifuged for 5 min at 300*g* and 4°C. The supernatant was transferred into a new reaction tube, supplemented with 0.1 volumes of 500 mM tris/HCl (pH 7.5) and 1.5 M NaCl, complemented with 10× protease inhibitor (Roche cOmplete Protease Inhibitor without EDTA; Roche Diagnostics), and again centrifuged for 15 min at 20,000*g* and 4°C. For cell lysis, cells were washed twice with ice-cold PBS, and subsequently, 0.5 ml of 1× radioimmunoprecipitation assay (RIPA) lysis buffer [50 mM tris/HCl (pH 7.5), 150 mM NaCl, 1% NP-40, 0.5% deoxycholate (DOC), and 0.1% SDS] supplemented with 1× protease inhibitor was added to each well. After 20 min, cells were scraped off and centrifuged for 15 min at 20,000*g* and 4°C. For further analysis, 0.2 volumes of 5× Laemmli buffer supplemented with 10% β-mercaptoethanol (β-Me) were added to the samples, which were then heated at 95°C for 5 min.

### Immunoblots and co-IP experiments

Protein samples were separated by SDS–polyacrylamide gel electrophoresis (SDS-PAGE) on 12% SDS-PAGE gels at 100 V for 2 hours and subsequently transferred to polyvinylidene difluoride membranes by blotting overnight at 30 V and 4°C. Thereafter, membranes were blocked for 3 hours at room temperature with tris-buffered saline containing skim milk powder and Tween 20 [MTBST; 25 mM tris/HCl (pH 7.5), 150 mM NaCl, 5% (w/v) skim milk powder, and 0.05% (v/v) Tween 20]. Binding of the primary antibody was carried out overnight at 4°C with anti–IL-12α (1:500 in MTBST; Abcam, ab133751), anti-IL-12β (1:500 in MTBST; Abcam, ab133752), EBI3 antisera (1:20 in PBS; provided by O. Devergne), anti-IL-27 (1:200 in MTBST; R&D Systems, AF2526), anti-HA tag (1:1000 in MTBST; BioLegend, 902301), anti-Hsc70 (1:1000 in MTBST; Santa Cruz Biotechnology, sc-1059), or anti-His tag (1:1000 in MTBST; Proteintech, HRP-66005). After washing, membranes were incubated for 1 hour in species-specific horseradish peroxidase–conjugated secondary antibodies (1:10,000 in MTBST; Santa Cruz Biotechnology). Immunoblots were detected with Amersham ECL prime (Cytiva) and a Fusion Pulse 6 imager (Vilber Lourmat).

For co-IP experiments, cells were lysed after washing with PBS in 0.5 ml of Triton lysis buffer [50 mM tris/HCl (pH 7.5), 150 mM NaCl, 1 mM EDTA, 1% Triton X-100, and 1× protease inhibitor] and cleared by centrifugation at 20,000*g* for 15 min and 4°C. To analyze secreted proteins, the medium was treated as described above and then precleared by the addition of 30 μl of Protein A/G agarose (Santa Cruz Biotechnology) and rotation for 1 hour at 4°C. Subsequently, all samples were incubated with 25 μl of target- specific magnetic beads [anti-FLAG M2 (Sigma-Aldrich, M8823) or anti-HA (Thermo Fisher Scientific, 88837)] while rotating for 2 hours at 4°C. Beads were washed three times with NP-40 wash buffer [50 mM tris/HCl (pH 7.5), 400 mM NaCl, 0.5% NP-40, and 0.5% DOC]. By adding 2× Laemmli buffer containing 4% β-Me and heating for 5 min at 95°C, proteins were eluted and then separated by SDS-PAGE.

For co-IP experiments with purified proteins, 1 μg of IL-12α^C96S,His^ and an equimolar amount of IL-12β^C199S^ (previously purified in our laboratory), 1 μg of EBI3 and equimolar mIL-27α (R&D Systems, 7430-ML-010), or 1 μg of IL-12α^C96S,His^ and equimolar EBI3 were mixed to a final volume of 200 μl (PBS) and incubated for 1 hour at room temperature. Ni–nitrilotriacetic acid beads (30 μl; Sigma-Aldrich) were added to the solution and incubated while rotating for 2 hours at 4°C. Beads were washed with PBS containing 20 mM imidazole, and elution was performed by the addition of PBS supplemented with 500 mM imidazole and incubation for 10 min at 4°C. For further analysis by SDS-PAGE, 0.2 volumes of 5× Laemmli buffer supplemented with 10% β-Me were added to the supernatants, which were then heated at 95°C for 5 min.

### Mammalian protein production and purification

For expression of IL-12α^C96S,His^, ExpiCHO cells (Thermo Fisher Scientific) were cotransfected with IL-12α^C96S,His^ and IL12β^C199S^ in a DNA ratio of 1:1 according to the manufacturer’s protocol (high titer). After protein expression for 7 days, the medium was centrifuged (at 5000*g* for 30 min at 4°C) and applied to a HisTrap HP column (Cytiva). A guanidinium chloride gradient (final 2.5 M GdmCl) was used to separate IL-12β^C199S^ from IL-12α^C96S,His^; subsequent washing with PBS was applied to refold IL-12α^C96S,His^ on the column. Elution was performed in PBS supplemented with 500 mM imidazole, and the His-tag was optionally cleaved by the addition of TEV protease [TEV: IL-12α^C96S^ 1:10 (w/w)] overnight at 4°C and removed by a second HisTrap HP column in PBS. Final purification was performed using a HiLoad 26/600 Superdex 200 pg column (Cytiva) in PBS. EBI3 was cotransfected with IL27α^L162C,His^ into Expi293 (Thermo Fisher Scientific) cells with a DNA ratio of 1:1 according to the manufacturer’s protocol. After 2 days, the medium was centrifuged and applied to a HisTrap column. To separate the complex, a guanidinium chloride wash gradient (final 6 M GdmCl) was used to elute EBI3 from the column, which was further purified by a HiPrep 16/60 Sephacryl S-200 HR in PBS supplemented with 3 M GdmCl. EBI3 containing fractions were pooled, concentrated, and dialyzed against PBS for refolding.

### Far-UV CD spectroscopy

Measurements were performed on a J-1500 CD spectrometer (Jasco) using a 10 μM protein solution in PBS in a quartz cuvette with 1-mm path length. Spectra were recorded from 200 to 260 nm at 20°C, temperature transitions from 20° to 90°C at 222 nm for IL-12α^C96S^, and 218 nm for EBI3 with a heating rate of 30°C/hour.

### Hydrogen deuterium exchange mass spectrometry

Hydrogen deuterium exchange (HDX) measurements were performed following the protocols established in (*59*) using an ACQUITY UPLC M-class system equipped with automated HDX technology followed by an in-line Synapt G2-S QTOF HDMS mass spectrometer (Waters). In short, HDX kinetics were recorded for biological duplicates as technical triplicates tracking data points at 0 s, 10 s, 1 min, 10 min, 30 min, and 2 hours. At each data point, 3 μl of a 20 μM protein solution containing both proteins in molar 1:1 ratio was diluted automatically 1:20 with PBS buffer (pH 7.4) containing 99.9% D_2_O or H_2_O at each time point. The exchange was stopped by the addition of 1:1 quenching buffer [200 mM Na_2_HPO_4_, 200 mM KH_2_PO_4_ (pH 2.3), containing 4 M GdmCl and 200 mM TCEP] at 1°C. Proteolytic on-column digestion was performed on a Waters Enzymate BEH Pepsin Column (2.1 mm by 30 mm) at 20°C. The resulting peptides were separated by reverse-phase chromatography at 0°C using a Waters Acquity UPLC C18 1.7 μm Vangard 2.1 mm–by–5 mm trapping-column and a Waters Aquity UPLC BEH C18 1.7 μm, 1 mm–by–100 mm separation column applying an H_2_O to acetonitrile gradient with both eluents containing 0.1% formic acid (v/v) to allow deuterium replacement with hydrogen from side chains and N/C termini that exchange faster than backbone amide linkages. Mass spectrometry data of eluting peptides were collected over a mass/charge ratio range of 100 to 2000 using Glu-fibrino peptide B (Waters) to ensure mass accuracy. Peptides were identified by MS^E^ ramping the collision energy automatically from 20 to 50 V. Because of the use of an automated system, deuterium levels were not corrected for back exchange and are reported as relative levels. Data analysis was performed with the PLGS (version 3.0.3) and DynamX (version 3.0) software packages (Waters).

### Analytical ultracentrifugation

Sedimentation velocity analytical ultracentrifugation (SV-AUC) experiments were performed on a Beckman Coulter Optima AUC analytical ultracentrifuge (Beckman Coulter) equipped with absorbance optics. For each sample, 350 μl of 10 μM IL-12α^C96S^ and EBI3 or the equimolar incubated proteins in PBS (pH 7.4) were loaded into a standard 12-mm double-sector epon-filled centerpiece, covered with quartz windows, alongside with 450 μl of the reference buffer solution. Samples were centrifuged at 42,000 rpm using an An-50 Ti rotor at 20°C (with an initial test run at 3000 rpm). Radial absorbance scans were acquired continuously at 235 nm with a radial step size of 0.001 cm. The obtained sedimentation velocity profiles were analyzed using SEDFIT software with a non–model-based continuous Svedberg distribution method [*c*(*s*)] (*66*). The density (ρ) and viscosity (η) of PBS used for data analysis was experimentally determined.

### NanoBRET assay

Receptor chains were cloned into the pHTC HaloTag CMV-neo Vector (IL-12Rβ2 and gp130) or the pNLF1-C [CMV/Hygro] Vector (IL-12Rβ2 and IL-27Rα) (Promega). COS-7 cells were cultivated under the same conditions as described for HEK293T cells, and transient transfections were performed in uncoated six-well plates using GeneCellin according to the manufacturer’s protocol. In total, 2 μg of DNA were transfected per well with a ratio of 100:1 HT:NL. After 16 hours, transfected cells were detached using Accutase (Sigma-Aldrich), divided into two pools, and 1 μl of HaloTag NanoLuc 618 Ligand (Promega) or 1 μl of dimethyl sulfoxide as a control per milliliters of cells were added. Cells (2 × 10^4^) were seeded into white-bottom 96-well plates and incubated for another 20 hours. Reconstituted proteins were incubated for 1 hour at room temperature, and cytokines were added for 30 min at a final concentration of 10 nM before measurement on a plate reader (BMG Labtech CLARIOstar). For inhibition experiments 1, 10, or 100 nM IL-12α^C96S^/IL-12β^C199S^/EBI3 was added 30 min before stimulation with 1 nM IL-12 or IL-27. Stimulation with IL-12α^C96S^/EBI3 (1 nM) or IL-12β^C199S^ (10 nM) were used as controls. Mean milliBRET units were calculated by dividing the acceptor emission by the donor emission and multiplication with 1000. To determine the mean NanoBRET ratio, the no-acceptor control mean is subtracted from the experimental mean. Samples are measured in technical triplicates, and one representative result from at least biological triplicates is shown.

### STAT assays

BL-2 cells were cultivated in RPMI 1640 (American Type Culture Collection modification, Thermo Fisher Scientific) supplemented with 20% heat-inactivated FBS (Gibco) and 1% (v/v) antibiotic-antimycotic solution [amphotericin B (25 μg/ml), streptomycin (10 mg/ml), and 10,000 U of penicillin; Sigma-Aldrich]. Cells were starved overnight in RPMI 1640 without FBS and antibiotic-antimycotic solution, and 1 × 10^6^ to 2 × 10^6^ cells in RPMI 1640 + 0.5% (w/v) bovine serum albumin (BSA; Sigma-Aldrich) were then seeded into 48-well plates and incubated for 15 min at 37°C. Reconstituted proteins were incubated for 1 hour at room temperature, and BL-2 cells were stimulated with cytokines for 1 hour at a final concentration of 10 ng/ml. For inhibition experiments, EBI3 (10, 100, or 1000 ng/ml) was added 30 min before stimulation with IL-27. Overnight treatment was performed with EBI3 at 10 ng/ml, and EBI3 at 10 ng/ml was added 30 min before IL-27 stimulation. Ice-cold PBS + 0.05% NaN_3_ (600 μl) was added to each well to stop the reaction, and the cells were transferred into a reaction tube, which was then centrifuged at 300*g* for 5 min at 4°C. Supernatant was discarded, and cells were lysed by the addition of 100 μl of NP-40 buffer supplemented with 1× protease inhibitor and phosphatase inhibitor (Serva) and incubated for 20 min at 4°C while rotating. After centrifugation for 5 min at 20,000*g* and 4°C, 0.2 volumes of 5× Laemmli buffer supplemented with 10% β-Me were added to the supernatant, boiled for 5 min, and further analyzed via immunoblots using STAT1 (1:1000 in 5% BSA-TBST; Cell Signaling, 9172) or P-STAT1 (Tyr^701^) [1:1000 in 5% BSA-TBST; Cell Signaling, (58D6) 9167].

NK-92 cells were cultivated at 37°C and 5% CO_2_ in minimum essential medium (MEM) α (Sigma-Aldrich), containing NaHCO_3_ (2.2 g/liter). The medium was further supplemented with 0.2 mM myo-inositol (Sigma-Aldrich, I-7508), 0.1 mM β-Me, 0.02 mM folic acid (Sigma-Aldrich, F-8758), 12.5% FBS (Gibco), 12.5% horse serum (Thermo Fisher Scientific, 16050122), and freshly added IL-2 (100 U/ml; PeproTech, 200-02). NK-92 cells were starved overnight in medium without serum and IL-2. Cells (0.5 × 10^6^ to 1 × 10^6^) were seeded into 48-well plates and incubated at 37°C for 15 min before stimulation with cytokines (reconstituted proteins were previously incubated for 1 hours at room temperature). Stimulation was performed for 30 min at a final concentration of 10 ng/ml. For inhibition experiments, IL-12α^C96S^ or IL-12β^C199S^ at 10, 100, or 1000 ng/ml was added 30 min before stimulation with IL-12 (10 or 1 ng/ml). Overnight treatment was performed with IL-12α^C96S^ (10 ng/ml), and IL-12α^C96S^ (10 ng/ml) was added 30 min before IL-12 stimulation. Cells were harvested on ice, transferred into a reaction tube, and centrifuged at 300*g* for 5 min at 4°C. The cell pellet was reconstituted in 100 μl of RIPA lysis buffer supplemented with 1× protease and phosphatase inhibitor, and lysis was performed for 20 min at 4°C while rotating. Cell debris was removed by centrifugation at 20,000g for 5 min at 4°C, and the supernatant was supplemented with 0.2 volumes of 5× Laemmli buffer supplemented with 10% β-Me, heated for 5 min at 95°C, and further analyzed via immunoblots using STAT4 (1:1000 in 5% BSA-TBST; Cell Signaling, C46B10) or P-STAT4 (Tyr^693^) [1:1000 in 5% BSA-TBST; Cell Signaling, (D2E4) 4134].

PBMCs were cultivated in RPMI 1640 (Thermo Fisher Scientific) supplemented with 10% FBS (BioSell), gentamycin (1 μg/ml; Thermo Fisher Scientific), penicillin-streptomycin (100 U/ml; Thermo Fisher Scientific), and 2 mM l-glutamine (Thermo Fisher Scientific). Cells (1 × 10^6^) were seeded into 48-well plates and stimulated for 1 hour with HDM (10 μg/ml; Citeq Biologics) alone or in combination with EBI3 (10 ng/ml), IL-12α^C96S^ (10 ng/ml), IL-10 (10 ng/ml; Miltenyi Biotec), IL-27 (10 ng/ml; Peprotech), or IL-35 (10 ng/ml; Miltenyi Biotec). Ice-cold PBS (600 μl) was added to each well to stop the reaction. The plate was centrifuged at 300*g* for 5 min at 4°C. Supernatant was discarded, and cells were lysed by the addition of 100 μl of NP-40 buffer supplemented with 1× protease inhibitor and phosphatase inhibitor (Roche) and incubated for 20 min at 4°C while shaking. Lysate was transferred into a reaction tube, centrifuged for 5 min at 20,000*g* and 4°C, and 0.2 volumes of 5× Laemmli buffer + 10% β-Me were supplemented. The protein solution was boiled for 5 min and further analyzed via immunoblots.

### PBMC and monocyte-derived macrophage culture and flow cytometry analysis

PBMCs of healthy individuals were isolated and resuspended in RPMI 1640 (Thermo Fisher Scientific) supplemented with 10% FBS (BioSell), gentamycin (1 μg/ml; Thermo Fisher Scientific), penicillin-streptomycin (100 U/ml; Thermo Fisher Scientific), and 2 mM l-glutamine (Thermo Fisher Scientific). Cells (1 × 10^6^) were seeded into 24-well plates and stimulated for 24 hours with LPS (100 ng/ml; InvivoGen) or HDM (10 μg/ml; Citeq Biologics) and additionally with EBI3 (10 ng/ml), IL-12α^C96S^ (10 ng/ml), IL-10 (10 ng/ml; Miltenyi Biotec), IL-27 (10 ng/ml; Peprotech), IL-35 (10 ng/ml; Miltenyi Biotec), or the combination of both IL-12α^C96S^ and EBI3. Stimulation of polarized PBMCs with EBI3 (10 ng/ml), IL-12α^C96S^ (10 ng/ml), IL-10 (10 ng/ml), IL-27 (10 ng/ml), and IL-35 (10 ng/ml) was done after 24 hours of preincubation with human IL-4 (10 ng/ml; Miltenyi Biotec). PBMCs were also used for CD14 MACS seperation, generating monocyte-derived macrophages (MDMs) from the CD14^+^ fraction as previously reported (*67*, *68*). A total of 0.5 × 10^6^ cells/ml were cultured in RPMI 1640 (Thermo Fisher Scientific) supplemented with 10% FBS (BioSell), gentamycin (1 μg/ml; Thermo Fisher Scientific), penicillin-streptomycin (100 U/ml; Thermo Fisher Scientific), 2 mM l-glutamine (Thermo Fisher Scientific), human GM-CSF (10 ng/ml; Miltenyi), and human TGF-β (2 ng/ml; Miltenyi) for 6 days at 37°C with 5% CO_2_ to differentiate the cells into alveolar-like macrophages (*48*). After 6 days of incubation, MDMs were harvested, 1 × 10^5^ to 2 × 10^5^ cells were seeded into 96-well plates and stimulated for 24 hours with HDM (10 μg/ml) and additionally with EBI3 (10 ng/ml), IL-12α^C96S^ (10 ng/ml), the combination of both IL-12α^C96S^ and EBI3, or IL-35. During harvest, supernatants were stored at −70°C until further cytokine analysis, and cells were lysed in RLT buffer (QIAGEN) supplemented with 1% β-Me and stored at −70°C until RNA isolation.

For T_reg_ induction and characterization, 2 × 10^5^ PBMCs per well were resuspended in RPMI 1640 medium (Thermo Fisher Scientific) supplemented with 10% heat-inactivated and filtered FBS (Sigma-Aldrich) and 1% penicillin-streptomycin (Thermo Fisher Scientific) and incubated with EBI3, IL-12a^C96S^ or PBS at 10, 20, 50 or 100 ng/ml as control for 72 hours at 37°C in a 5% CO_2_ atmosphere. T_reg_ induction was characterized as CD3^+^CD4^+^CD127^−^CD25^hi^FoxP3^+^ cells by fluorescence-activated cell sorting as previously described (*69*) with the following anti-human antibodies and clones: CD3 (clone UCHT1), CD4 (clone RPA-T4), CD127 (clone A019D5), and CD25 (clone BC96) (all from BioLegend) and FoxP3 (clone PCH101) (Invitrogen). For SEA stimulation, 2 × 10^5^ PBMCs were left untreated (PBS control) or cultured with SEA (50 μg/ml), prepared from *S. mansoni* eggs as previously detailed (*70*), alone or in combination with either EBI3 or IL-12α^C96S^ at 10 ng/ml for 5 days. On day 3, 50% of culture medium was exchanged with fresh medium containing respective stimuli (SEA ± EBI3 or IL-12^C96S^) or PBS as control. Culture supernatants were collected, and IL-4 concentrations were determined by enzyme-linked immunosorbent assay (ELISA).

### Cytokine analysis (ELISA)

PBMC supernatants were analyzed for IL-4 using the IL-4 Human ELISA Kit from Thermo Fisher Scientific (KHC0041) and for CCL17 using the human CCL17/TARC DuoSet ELISA Kit from R&D systems (DY364). For both PBMC and MDM supernatants, IL-6, IL-1β, and IL-8 secretion using the human ELISA sets from BD Biosciences (555220, 557953, and 555244) and TNF-α secretion using the human DuoSet ELISA (R&D Systems, DY210) were analyzed. All ELISAs were performed according to the manufacturer’s instructions.

### RNA isolation

RNA was extracted using a spin-column kit according to the manufacturer’s instructions (Zymo Research) and transcribed into DNA using the High-Capacity cDNA Reverse Transcription Kit according to the manufacturer’s instructions (Applied Biosystems).

### Reverse transcription quantitative polymerase chain reaction

cDNA (10 ng) was used as a template, and primers were mixed with FastStart Universal SYBR Green Master Mix (Roche). Fluorescence was measured on a ViiA7TM Real-Time PCR System (Applied Biosystems, Thermo Fisher Scientific). The expression levels were normalized to the house-keeping genes *GAPDH* (for MDM), *ACTB*, and 18*S* (for PBMCs). Relative gene expression was calculated as 2ΔCT [ΔCT = CT(Housekeeper) − CT(Gene)]. Primer sequences for RT qPCR are shown in Table 1.

**Table 1.** Primer sequences used for quantitative polymerase chain reaction.

Human primer	Forward sequence	Reverse sequence
*18S*	GTAACCCGTTGAACCCCATT	CCATCCATTCGGTAGTAGCG
*ACTB*	GGATGCAGAAGGAGATCACT	CGATCCACACGGAGTACTTG
*CXCL8*	GAAGTTTTTGAAGAGGGCTGAGA	TGCTTGAAGTTTCACTGGCAT
*GAPDH*	GAAGGTGAAGGTCGGAGT	GAAGATGGTGATGGGATTTC
*IL1B*	AGAAGTACCTGAGCTCGCCA	CTGGAAGGAGCACTTCATCTGT
*IL6*	ACATGTGTGAAAGCAGCAAAG	GGCAAGTCTCCTCATTGAATCC
*TNF*	CCCATGTTGTAGCAAACCCTC	TATCTCTCAGCTCCACGCCA
*TGFB*	CACGCAGTACAGCAAGGTCC	CCACGTAGTACACGATGGGC
*IL5*	TCTCCAGTGTGCCTATTCCC	CGAACTCTGCTGATAGCCAA

### Structural modeling

We generated a model structure for the IL-35 sequence with the AlphaFold multimer module (*71*, *72*). The sequences of human IL-12α and EBI3 without the signal-peptide were used as input, and the best-ranked structure from the output was taken as the final model structure.

### Quantification and statistics

Immunoblots were quantified using the Bio-1D software (Vilber Lourmat). Statistical analyses were performed using Prism (GraphPad Software). Differences were considered statistically significant when *P* < 0.05. Where no statistical data are shown, all experiments were performed at least three times, with one representative experiment depicted in figures. For immunological data, Friedman test was used and *P* < 0.05 was considered statistically significant. Details of statistical tests and sample sizes are provided in the figure legends.
