# Supplementary material for: Human interleukin-12α and EBI3 are cytokines with anti-inflammatory functions
Source: Sci Adv. 2023 Oct 25;9(43):eadg6874. doi: 10.1126/sciadv.adg6874 (PMC10599630; doi:10.1126/sciadv.adg6874)
Supplement: Supplementary file 1 — Figs. S1 to S6 [file sciadv.adg6874_sm.pdf]

Science Advances
AAAS

Supplementary Materials for

Human interleukin-12α and EBI3 are cytokines with anti-inflammatory functions

Karen Hildenbrand *et al.*

Corresponding author: Matthias J. Feige, matthias.feige@tum.de

Sci. Adv. **9**, eadg6874 (2023)
DOI: 10.1126/sciadv.adg6874

This PDF file includes:

Figs. S1 to S6

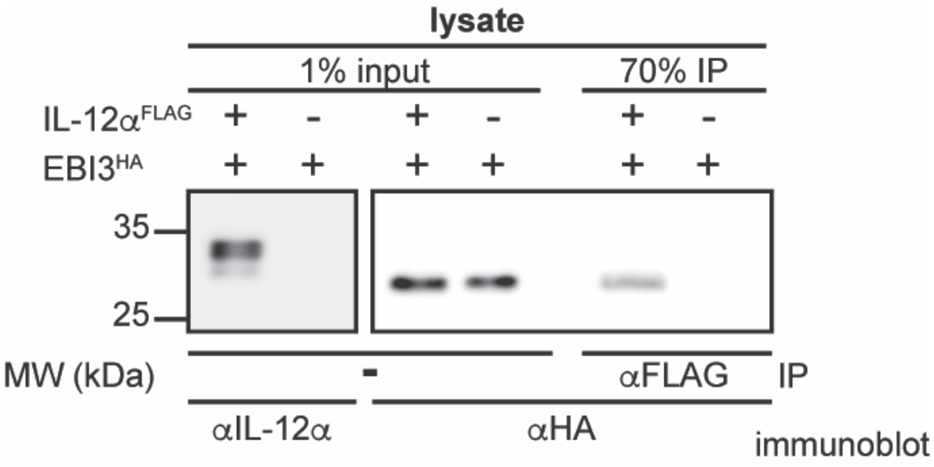

Fig. S1.

Interaction of IL-12α and EBI3. Co-immunoprecipitation of HA-tagged EBI3 with FLAG-tagged IL-12α in cell lysates shows assembly for these two proteins. Constructs were expressed in HEK293T cells. One representative immunoblot is shown.

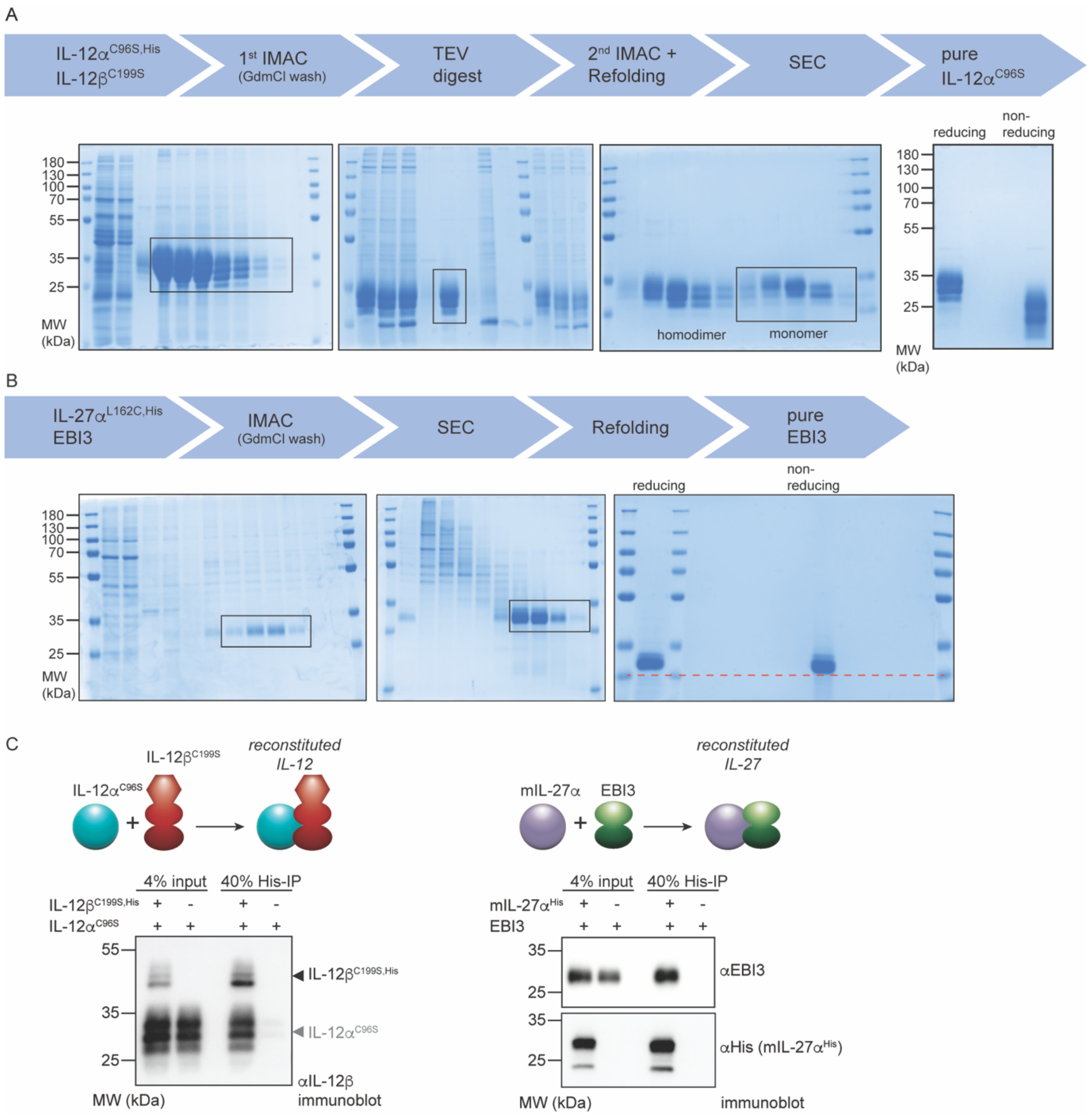

Fig. S2.

Recombinant IL-12α^{C96S} and EBI3 are pure and interaction-competent proteins. (**A**) Detailed purification strategy with the corresponding Coomassie stained SDS-gels of IL-12α^{C96S} and (**B**) EBI3. Pooled fractions are shown in rectangles. For EBI3, the red dashed line is shown to guide the eye for the comparison of its migration behavior under reducing *versus* non-reducing conditions. Note that the gel showing purified IL-12α^{C96S} (top right) is the same as in main Figure 3A. (**C**) Recombinant IL-12α^{C96S} and EBI3 interact with their partner subunits to form IL-12 and IL-27, respectively. IL-12α^{C96S} and IL-12β^{C199S,His} or EBI3 and murine IL-27α^{His} were incubated and co-immunoprecipitation using the His-tag which reveals specific interaction. Murine IL-27α was used for all experiments were needed since human IL-27α cannot be produced in isolation.

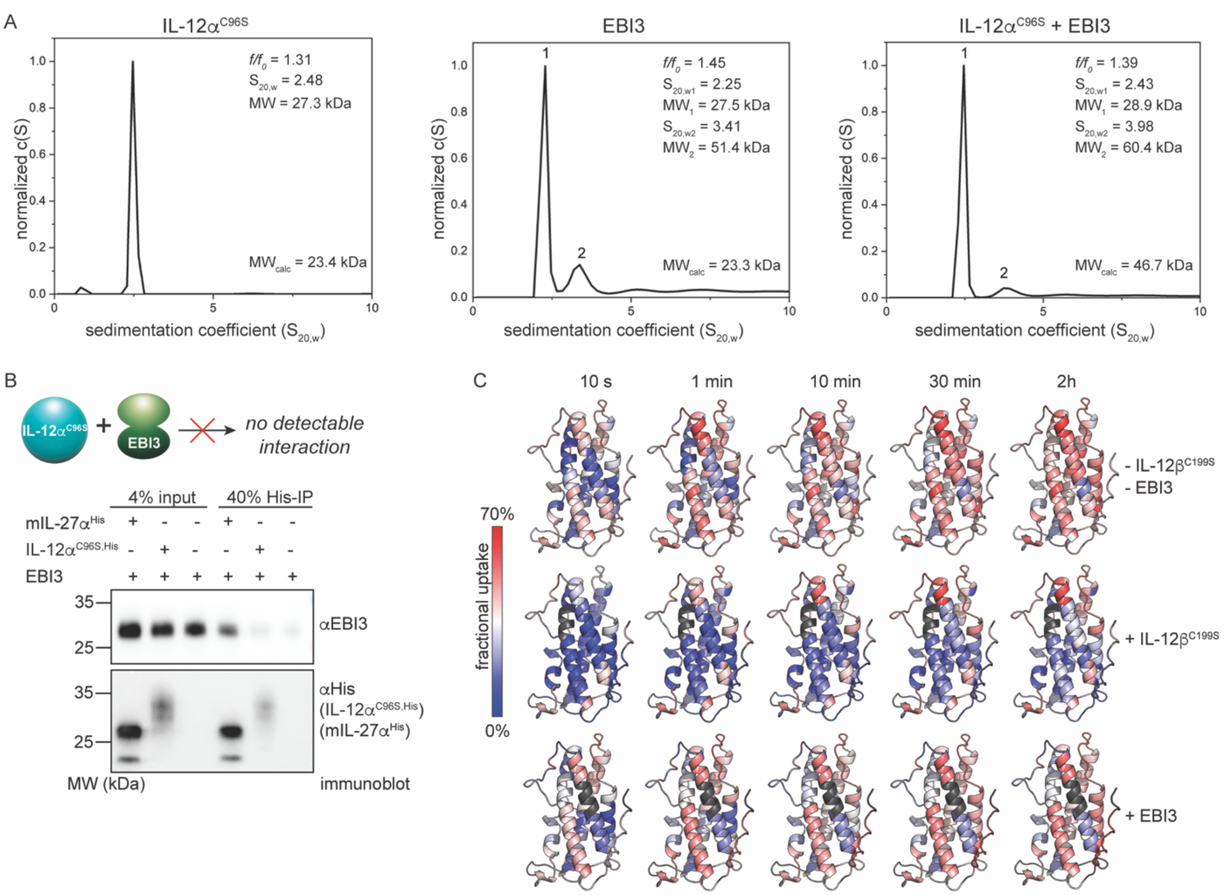

Fig. S3.

Recombinant IL-12α^{C96S} and EBI3 do not detectably interact to form IL-35. (**A**) Analytical ultracentrifugation experiments of IL-12α^{C96S} and EBI3 confirm a mostly monomeric state of both proteins, with frictional ratios of 1.31 (IL-12α^{C96S}) and 1.45 (EBI3) and calculated molecular weights for the monomers of 27.3 kDa (IL-12α^{C96S}) and 27.5 kDa (EBI3). Some homodimers (51.4 kDa) are detected for EBI3. Incubation of both proteins did not indicate the formation of a heterodimeric complex. (**B**) Individually purified IL-12α^{C96S,His} and EBI3 do not co-immunoprecipitate in contrast to EBI3 and mIL-27α^{His}. (**C**) Hydrogen/deuterium exchange (HDX) experiments reveal IL-12α^{C96S} stabilization through complex formation only after incubation with IL-12β^{C199S} in contrast to EBI3. IL-12α^{C96S} is colored according to the fractional uptake of HDX measurements. Blue color indicates a low (less flexible and potentially shielded regions) and red colors a high (flexible and solvent accessible regions) fractional uptake (gray: no sequence coverage in HDX measurements).

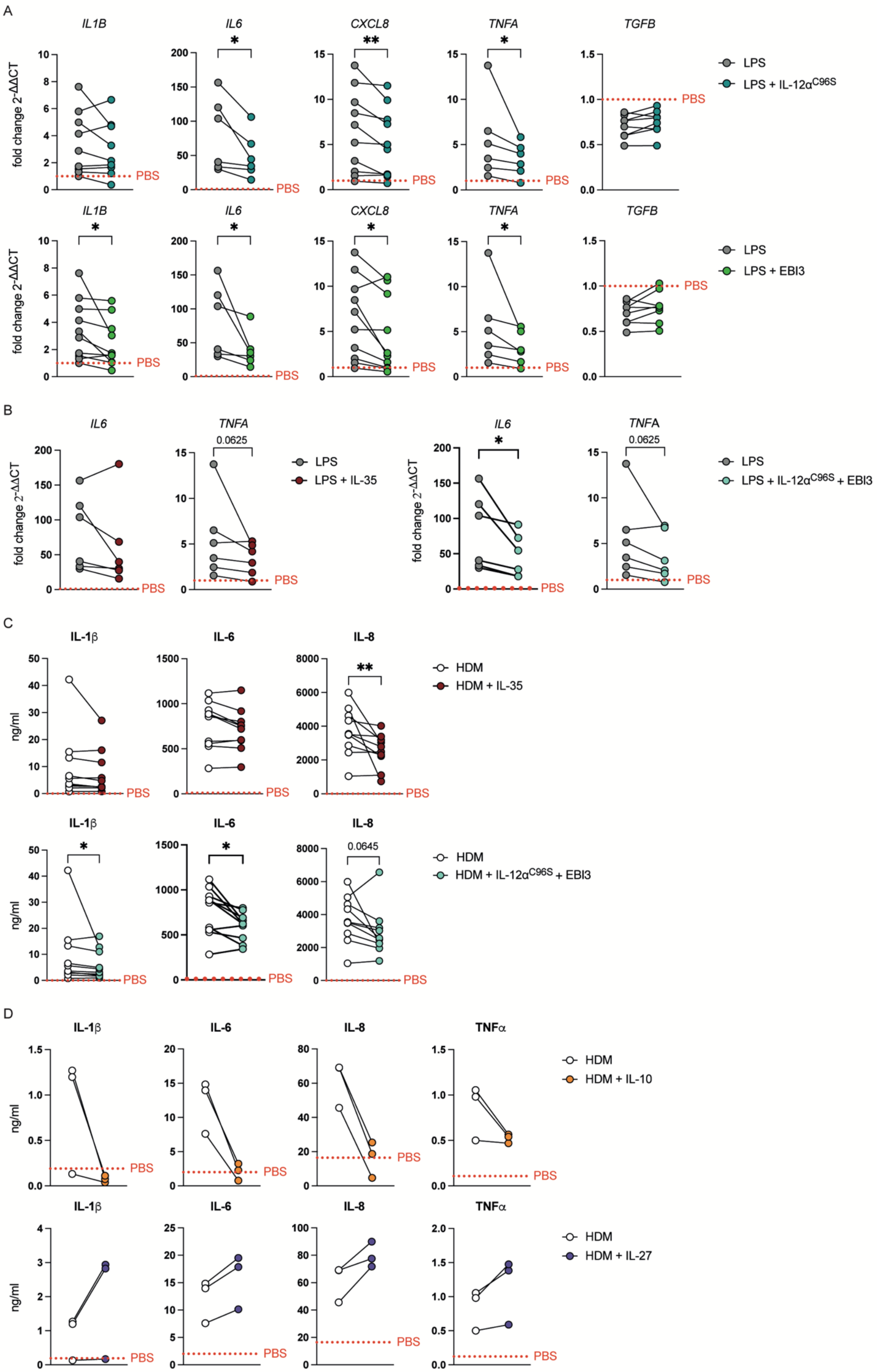
A
IL1B
IL6
CXCL8
TNFA
TGFB
fold change 2^-ΔΔCT
PBS
*
**
LPS
LPS + IL-12α^C96S
LPS + EBI3
B
IL6
TNFA
0.0625
LPS
LPS + IL-35
LPS + IL-12α^C96S + EBI3
C
IL-1β
IL-6
IL-8
ng/ml
0.0645
HDM
HDM + IL-35
HDM + IL-12α^C96S + EBI3
D
TNFα
HDM + IL-10
HDM + IL-27

Fig. S4.

Functional effects of IL-12^{C96S}, EBI3 and control cytokines. (**A**) Gene expression of *IL1B*, *IL6*, *CXCL8*, *TNFA* and *TGFB* (qPCR) from LPS-stimulated human PBMCs after additional treatment with IL-12^{C96S} or EBI3 (n = 6 to 10 donors). (**B**) Gene expression of *IL6* and *TNFA* (qPCR) from LPS-stimulated human PBMCs after additional treatment with IL-35 or the combination of IL-12a^{C96S} and EBI3 (n = 6 donors). (**C**) Concentrations of IL-1β, IL-6 and IL-8 (ELISA) in culture supernatants produced by human MDM (n = 10 donors) after stimulation with HDM alone or after additional treatment with IL-35 or the combination of IL-12a^{C96S} and EBI3. (**D**) Concentrations of IL-1β, IL-6, IL-8 and TNFα (ELISA) in culture supernatants produced by human PBMCs (n = 3 donors) after stimulation with HDM alone or after additional treatment with IL-10 or IL-27. Dotted line indicates mean secretion or gene expression of PBS treated PBMCs or MDMs. Data are presented as individual values. Donor dependent effect is shown by connecting line. Statistical significance was determined by Wilcoxon test. *$p< 0.05$; **$p < 0.01$.

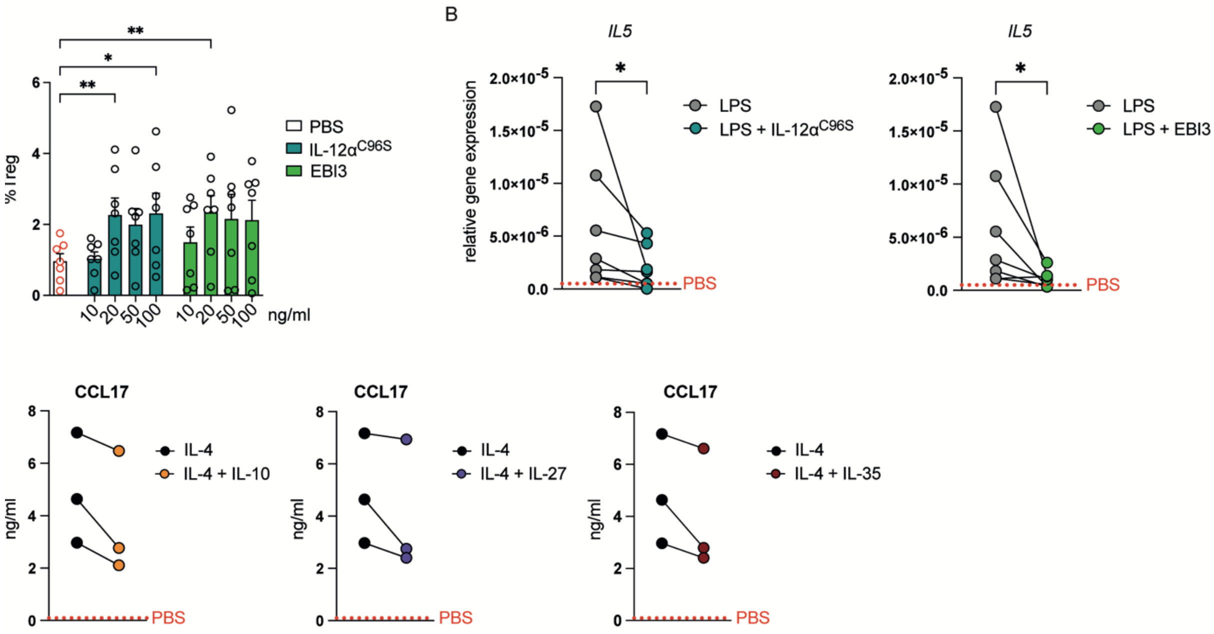

Fig. S5.

Effects of IL-12^{C96S}, EBI3 and control cytokines on Treg cells and type 2 immunity. (**A**) Percentage of CD25^{hi}Foxp3^{+} Treg cells (FACS) in human PBMC cultures treated with increasing concentrations of IL-12a^{C96S} or EBI3 (10-100 ng/ml) (n = 7). Data is presented as means + SEM. Statistical significance was determined by Friedmann test. *p < 0.05; **p < 0.01. (**B**) Gene expression of *IL5* (qPCR) from LPS stimulated human PBMCs after additional IL-12a^{C96S} or EBI3 treatment (n = 7 donors). (**C**) Concentration of CCL17 (ELISA) in culture supernatants produced by human PBMCs (n = 3 donors) after stimulation with IL-4 alone or after additional treatment with IL-10, IL-27 and IL-35. (**B-C**) Dotted line indicates mean secretion or gene expression of PBS treated PBMCs. Data are presented as individual values. Donor dependent effect is shown by connecting line. Statistical significance was determined by Wilcoxon test. *p< 0.05.

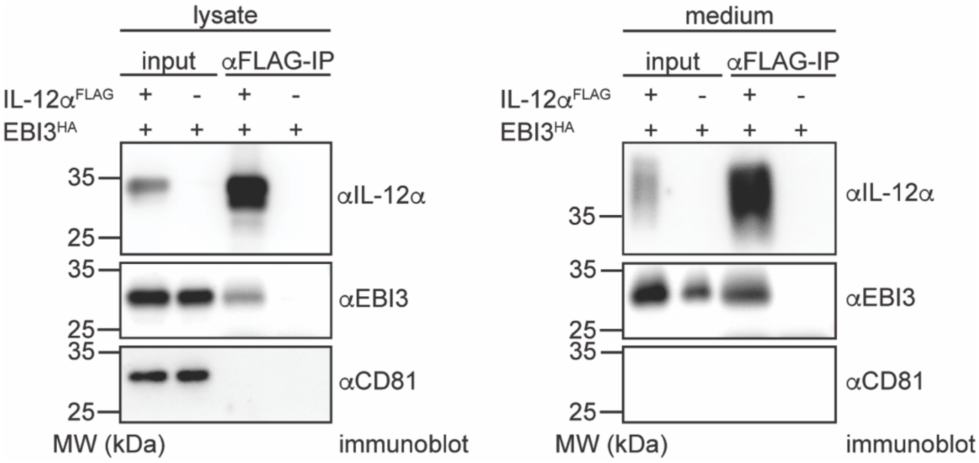

Fig. S6.

Testing CD81 interactions in IL-35. CD81 does not co-immunoprecipitate with IL-12α and EBI3. Co-immunoprecipitation of FLAG-tagged IL-12α with HA-tagged EBI3 in cell lysates and in the medium verifies assembly for these two proteins. Endogenous CD81 can be detected in the input lysate fraction only.
